# The Rule in Bathing

**Published:** 1915-06-19

**Authors:** 


### The Rule in Bathing.

The second lecture on "Water Cures" was delivered by Dr. Fortescue Fox at the Royal Society of Medicine on June 3. The special division of the subject dealt with in this lecture was the effect of cold and heat on the human body. The root of all water-cures was the conveyance to or abstraction from the body of heat; the whole organism responded to these impressions, and heat differences, within certain limits, had a profound effect on the vital processes. Man could endure great differences of temperature, and his capacity for generating heat was extraordinary; in twenty-four hours the heat engendered by him sufficed to raise 55 lbs. of water from the freezing to the boiling point. The loss of heat from the body was minimised by the non-conducting skin, which also acted in a way which entitled it to the term "sensitive plate"; it was constantly receiving and conveying impressions from the external world. The effects of cold were common knowledge. The average surface temperature of the human body was 93° Fahr.

For most persons a bath of 120° Fahr. was intolerably hot. Moist air was an admirable means of conveying heat. Baths might be regarded as artificial and temporary climates, and by their agency reactions could be produced in any part of the body as desired. On entering a cold bath an immediate contraction of the vessels ensued, producing a blanching of the skin, and in the deeper parts of the body there were corresponding changes; greater breathing became deeper, and there was a consequent greater intake of oxygen and a heightened blood-pressure. After a few moments the circulation returned to the skin as a sort of heat-wave, *i.e.*, the reaction, as shown by a pink colouration of the skin and an evident quickening in muscles and brain. But as cold bathing made a considerable demand on the nerve energy, the bather must be in fair general health. A cool bath without a reaction he stigmatised as a "physiological crime."

With regard to the effect of heat, during our winter campaign in Flanders nothing gave our soldiers greater relief than the hot baths provided for them; they proved a more powerful restorative factor than food. No people took such hot baths as the Japanese, but they finished with cold douching, and they were the most scientific bathers in the world. Their baths often reached 123° Fahr. "Cold after heat" was the golden rule for bathing, and should be exhibited in every bathroom, particularly in temperate climates.

